# Readiness towards online learning among physiotherapy undergraduates

**DOI:** 10.1186/s12909-021-02803-8

**Published:** 2021-07-10

**Authors:** Harikrishnan Ranganathan, Devinder Kaur Ajit Singh, Saravana Kumar, Shobha Sharma, Siew Kuan Chua, Nabilah Binti Ahmad, Kamalambal Harikrishnan

**Affiliations:** 1 Physiotherapy Programme, Centre for Healthy Ageing and Wellness, Faculty of Health Sciences, Universiti Kebangsaan Malaysia, Jalan Raja Muda Abdul Aziz, Kuala Lumpur, Malaysia; 2 Physiotherapy Programme, Faculty of Allied Health Sciences, University of Cyberjaya, Cyberjaya, Selangor Malaysia; 3 Allied Health and Human Performance, University of South Australia, Adelaide, Australia; 4 Speech Science Programme, Centre for Healthy Ageing and Wellness, Faculty of Health Sciences, Universiti Kebangsaan Malaysia, Jalan Raja Muda Abdul Aziz, Kuala Lumpur, Malaysia; 5 Centre of Physiotherapy, Faculty of Health Sciences, Universiti Teknologi MARA, Puncak Alam, Selangor, Malaysia; 6 Physiotherapy Programme, School of Health Sciences, KPJ Healthcare University College, Nilai, Malaysia; 7 Independent Researcher, Cyberjaya, Malaysia

**Keywords:** Online learning, Physiotherapy undergraduates, Readiness

## Abstract

**Background:**

Online learning is an attractive option for educators, especially as means of overcoming the challenges posed by the global pandemic. Although it is best to evaluate student readiness prior to commencement of an online course, to ensure successful development and delivery of student-centric teaching and learning strategies, readiness towards online learning among physiotherapy undergraduates is unknown. The main aim of this study was to examine physiotherapy undergraduates’ readiness towards online learning.

**Methods:**

In this cross-sectional study, participants were selected through a combination of total population and convenience sampling. The Student Online Learning Readiness questionnaire was distributed among physiotherapy undergraduates from two public and two private universities in Malaysia to investigate their technical, social and communication competencies. Information about device characteristics were obtained to evaluate their equipment readiness. Descriptive and group comparisons were conducted using independent t-test, and analysis of variance with *p* < 0.05 as level of significance.

**Results:**

A total of 352 physiotherapy undergraduates participated. The response rate was 81.6%. The results showed that physiotherapy undergraduates in these four institutions had moderate levels of readiness towards technical competencies (M = 3.7 ± 0.5), social competencies with instructor (M = 3.7 ± 0.6), social competencies with classmates (M = 3.8 ± 0.6) and communication competencies (M = 3.6 ± 0.5) related to online learning. The overall readiness for these four competencies was moderate (M = 3.7 ± 0.4), however the physiotherapy undergraduates had high (> 80% possessed smartphones and laptop) level of equipment readiness. Institution and gender had no significant effect on the level of readiness (*p* > 0.05). Year 1 and 2 had significantly higher levels of social competencies with instructor compared to final year physiotherapy undergraduates (*p* < 0.05).

**Conclusion:**

Physiotherapy undergraduates in these four institutions had moderate to high levels of readiness towards online learning. Technical, social and communication competencies could be further enhanced with appropriate strategies.

**Significance:**

This study provides an insight into the level of readiness towards online learning among physiotherapy undergraduates. The findings of our study shed light on issues to consider when designing online courses. A pre-course training for undergraduates prior to the commencement of online courses may be useful.

**Trial registration:**

Not Applicable.

## Background

Higher education including physiotherapy has been confronted by numerous challenges [1, 2]. These include a new generation of learners [3], pedagogical shift in learning [4], teaching of psychometric skills [5] and lack of standard instructional methods. Thus, online learning has been an attractive option for educators to address some of these challenges, more so with the current COVID-19 global pandemic. In fact, online learning is becoming mandatory [6] in many institutions.

Online learning can be defined as computer or smart phone-based instructions that students typically access over the internet, outside of the traditional classroom learning environments [7]. Online courses have been reported to have various advantages such as having greater learning flexibility, diverse representation of students [7], promoting collaboration, self-directed learning [8], crowd sourcing, overcoming the barriers of distance and time [9], as well as being able to attract expertise beyond geographical boundaries [10]. While online learning may enhance students’ cognitive reading skills, autonomy, and their motivation [11], these courses also report large dropout rates (70 to 90%) [12]. The exact reasons for this are not clear but could be attributed to the course not suiting the specific needs of the learners, unengaging course design or even a lack of readiness towards online learning.

Historically, many online courses have been mostly targeted at those who are already part of the workforce and postgraduates with prior knowledge in the field. The pedagogical approach for undergraduates has continued to rely on face to face mode [1]. Undergraduates who have just completed their school education and do not have any work experience may find it difficult to engage with, and learn from, online courses. Undergraduates can be considered as learners who are in the transition from pedagogical to andragogical model of instructions. Thus, the delivery of online courses to undergraduates may likely differ.

Readiness towards online learning may be critical in successful implementation of online learning [13, 14] among undergraduates. Although many studies have been undertaken among university students [15–23], there are limited studies conducted specifically among undergraduates to infer the readiness towards online learning ([13, 24–28] and [14]).

Within the Malaysian higher educational context, there has been some research pertaining to readiness for online learning [12, 29, 30], readiness for blended learning [24] and readiness for Massive Open Online Courses [12]. These studies however are not specifically focused on undergraduates and they evaluate varied competencies. Collectively, the findings indicate that students are only moderately ready. In most of the studies, technical competencies, or information and communication technology (ICT) skills [12, 24, 29] and equipment readiness [12, 30] have been evaluated. Other competencies that have been examined include self-directed learning attitudes [24], communication competencies, social competencies, and self-efficacy [12].

Understanding or analysing the learners’ needs is the first recommended step in developing an online course in instructional designs such as ASSURE [31] and ADDIE [32–34]. Kemp design models have also been used to determine learner characteristics as a component of instructional design process [35, 36]. However, the learner characteristics or their readiness for online courses among physiotherapy undergraduates has not been previously researched. Recognising student readiness upfront could encourage participation, engagement and hence facilitate effective learning [21]. Furthermore, while most research to date has focused on technical competencies, access to technology and self-directed learning, other competencies such as social and communication have been rarely evaluated [14]. Furthermore, there is also limited literature on online learning readiness among undergraduates from health science background where students are required to acquire psychomotor and cognitive skills. Therefore, the objective of this study was to determine physiotherapy undergraduates’ readiness for online learning in Malaysia.

## Methods

All physiotherapy undergraduates from four universities (two public and two private, *N* = 431) were invited to participate in this cross-sectional study. A combination of total population and convenience sampling was used to recruit participants for this study. Written participation permission was obtained from the Faculty Deans or Head of Programmes. Ethical approval was obtained from the Research Ethics Committee, Universiti Kebangsaan Malaysia (UKMPPI/III/8/JEP-2019-810).

The study used Student Online Learning Readiness (SOLR) questionnaire [14]. Prior to the commencement of this study, written permission was obtained from the developers of the questionnaire. The SOLR uses a 5-point Likert scale, which is considered as an ideal number for Likert scales [37]. The questionnaire consists of 20 questions including six questions on technical competencies, four questions on communication competencies, five questions on social competencies with instructor and five questions on social competencies with classmates. All four components have high reliability (Cronbach’s α > 0.823) and validity. Its exploratory factor analysis (EFA) shows that all four factor-structures of the instrument explain 66.69% of the variance in the pattern of relationships among the items [14]. The SOLR questionnaire also has acceptable level of construct validity which is identified using confirmatory factor analysis [38]. Additional information such as demographic data and device characteristics were also collected, which were used to infer equipment readiness.

The survey was conducted between November and December 2019. The undergraduates were provided documents electronically using a Google Form™ which included a consent form, personal information sheet (gender, year of study, nationality, devices owned) and the SOLR questionnaire. The questionnaire was forwarded to all physiotherapy undergraduates through student groups on a social media application (WhatsApp). Students were allocated 2 weeks to submit the completed questionnaire. To overcome the risk of missing data, all the fields within the Google Form were set in a manner that it had to be completed or answered (must provide value feature), in order for the respondent to move on to the next question. This ensured all fields would be completed and there would be no missing data.

An optimal response rate greater than 80% was targeted [39]. The risk of non-response and sampling bias were minimised by targeting a higher response rate [40] and including participants from several universities [14], respectively. The questionnaire was anonymised without participants’ names to ensure there was no social desirability bias. However, there is a possibility of bias when using self-assessment questionnaire [38].

### Data analysis

The data from the SOLR questionnaire were downloaded onto a Microsoft Excel™ worksheet and was analyzed using SPSS™ (version 22, IBM Corp., Armonk, New York). The undergraduates’ response to equipment readiness was evaluated by percentage of possession of devices.

Firstly, the average score for each competency for individual undergraduate was calculated and then the overall mean, median and standard deviation of the respective competencies were inferred. The overall readiness score was obtained through the mean value of responses for all questions in the SOLR questionnaire. The skewness and kurtosis values for all the competencies and variables such as gender, institution, year of study were below the threshold value [18] ±1 and ± 3, respectively. Hence parametric test was used to infer the difference in level of readiness among the variables. The difference in readiness between genders was analysed using independent t-test whereas differences in readiness among institutions and year of study were evaluated using one-way Analysis of Variance (ANOVA). The mean value of < 3 indicates low, 3 and < 4 is moderate and 4 and > 4 is considered as high level of readiness [26]. The level of significance set for this study is 0.05 with 95% as confidence interval.

## Results

A total of 352 physiotherapy undergraduates responded to the survey (response rate of 81.6%). Sociodemographic details of the participants are as shown in Table 1.

**Table 1:** Participants Socio-demographic information and online learning readiness data

Variable	
**Gender, n (%)**	
Male	61 (17.3)
Female	291 (82.7)
**Participants based on nationality, n (%)**	
Malaysian	336 (95.5)
Non-Malaysian	16 (4.5)
**Participants based on institution, n (%)**	
UNIVERSITY A	91 (25.9)
UNIVERSITY B	34 (9.7)
UNIVERSITY C	58 (16.5)
UNIVERSITY D	169 (48.0)
**Participants based on year of study, n (%)**	
1	84 (23.9)
2	87 (24.7)
3	95 (27.0)
4	86 (24.4)
**Possession of devices, %**	
Smart Phone	96.9
Laptop	83.1
Desktop	4
Tablet	6.8
**Overall readiness, mean (SD) /median/range**	3.7 (0.4) /3.7/2.7–5.0
**Competencies, mean (SD) /median/range**	
Technical Competencies	3.7 (0.5)/3.6/1.67–5.0
Social Competencies with Instructor	3.7 (0.6)/3.8/2.0–5.0
Social Competencies with Classmates	3.8 (0.6)/4.0/2.0–5.0
Communication Competencies	3.6 (0.5)/3.7/2.25–5.0

n: Frequency

*SD:* Standard Deviation

Undergraduates from year 1 to 4 were equally represented. 82% of the undergraduates were females. Most of the undergraduates were Malaysian citizens. Cronbach alpha (internal consistency) of the SOLR questionnaire was > 0.7 for overall items and individual factors. The overall mean and individual competencies readiness scores were 3 to 4, suggesting moderate level of readiness for online learning. All the competencies in the SOLR (Table 1) demonstrated moderate level of readiness, with highest score for social competencies with classmates (M = 3.8 ± 0.6) and least score for communication competencies (M = 3.6 ± 0.5).

Tables 2 and 3 highlight the role of gender, year of study, and type of institution, on readiness scores. There were no statistically significant differences (*p* > 0.05) in level of readiness in terms of gender and type of institution (public / private). There was an effect in level of social competencies with instructor based on year of study, F (3351) = 3.673, *p* = 0.012. Post hoc analyses with Tukeys HSD (Table 4) indicated that year 1 (M = 3.83, SD = 0.6) and year 2 (M = 3.81, SD = 0.43) physiotherapy undergraduates had higher readiness towards social competencies with instructor when compared to year 4 undergraduates (M = 3.57, SD = 0.61). However, there were no statistically significant differences (*p* > 0.05) for other competencies in the SOLR.

**Table 2:** Effects of gender and type of institution on online learning readiness competencies

Variable	Competencies	Mean Difference	95% Confidence Interval of difference		t	Sig (2-tailed)
			Lower	Upper		
**Gender**	TC	0.4924	−0.9830	0.1968	0.656	0.965
	SCWI	0.15471	−0.0041	0.3135	1.916	0.715
	SCWC	0.03237	−0.1390	0.2038	0.371	0.853
	CC	0.15292	0.0019	0.3039	1.991	0.052
**Type of institutions** (Public versus Private)	TC	−0.0294	−0.1461	0.0874	−0.494	0.621
	SCWI	0.0499	−0.0763	0.1760	0.406	0.437
	SCWC	−0.1070	−0.2421	0.0282	0.638	0.120
	CC	−0.0459	−0.1659	0.0742	0.864	0.453

*TC:* Technical competencies

*SCWI:* Social competencies with Instructor

*CC:* Communication competencies

*SCWC:* Social competencies with Classmates

t: Value of independent sample t-test

*Sig:* Level of significance

**Table 3:** Effects of institution and year of study on online learning readiness competencies

Variable	Competencies	Mean Square	f	Sig (2-tailed)
**Between the institutions**	TC	0.084	0.295	0.829
	SCWI	0.472	1.430	0.234
	SCWC	0.415	1.087	0.355
	CC	0.382	1.278	0.282
**Year of study**	TC	0.061	0.215	0.886
	SCWI^a^	1.190	3.673	0.012*
	SCWC	0.777	2.053	0.106
	CC	0.170	0.565	0.638

(*) *p* < 0.05

^a^ Post hoc analysis is tabulated in Table 4

*TC:* Technical competencies

*SCWI:* Social competencies with Instructor

*CC:* Communication competencies

*SCWC:* Social competencies with Classmates

*Sig:* Level of significance

f: Value of one-way Analysis of Variance (ANOVA)

**Table 4:** Multiple comparison of SCWI based on Tukey’s post -hoc analysis

Group comparison	Mean Difference	95% confidence intervals		Sig
		Lower	Upper	
YEAR 1–2	0.0070	−0.2077	0.2417	0.997
YEAR 1–3	0.1167	−0.1033	0.3368	0.519
YEAR 1–4	0.2564	0.0310	0.4818	0.018*
YEAR 2–3	0.0997	−0.1183	0.3177	0.639
YEAR 2–4	0.2394	0.0160	0.4628	0.030*
YEAR 3–4	0.1397	0.0790	0.3584	0.352

(*) *p* < 0.05

*SCWI:* Social competencies with Instructor

*Sig:* Level of significance

## Discussion

With online learning gaining increasing prominence, it is important to ensure the principles and process which underpin this pedagogical approach are robust. However, to date, there has been limited research, especially in developing countries such as Malaysia, which have explored if students are indeed ready to engage with online and if so, what factors influence this engagement. This research aimed to address this knowledge gap. The findings from this study highlight that undergraduate physiotherapy students do have moderate level of readiness for online learning. The standard deviation for all competencies was small (< 0.7) indicating minimal variability within the data [41], despite differences in year of study and institution. These findings build on evidence from previous studies conducted among students in higher educational institutions locally [12, 29, 30]. A recent study with undergraduates from three Malaysian institutions using the SOLR questionnaire inferred similar results for all the four competencies [42].

An interesting finding from this study indicates that readiness towards online learning has remained the same, or only slightly improved, compared to previous studies. A possible reason could be lack of web-based course familiarity previously (at pre-university level) which could be a substantial predictor for online readiness [18]. Previously, Asian learners were reported to prefer traditional classroom and were more inclined towards practicing their receptive rather than conversational skills [30]. This could also be another possible reason for the finding of moderate communication and social competencies in this research. Similarly, previous research indicated that some educators, who were from conventional schools, reported not to be ready for the transition to online learning [30]. In a study at 27 Malaysian higher educational institutions, 1635 lecturers revealed that about 30% of them preferred traditional teaching methods [43]. The readiness of the educator could have an impact on learners [44]. However, given the COVID-19 pandemic and the entire education sector’s move towards online learning, it is unclear if these reasons continue to be influencing factors in the current context. Furthermore, this study did not collect any information on the readiness among educators, which is a limitation of this research.

The findings from the current study suggest that Malaysian undergraduates’ online readiness is mid-range when compared to similar studies from other geographical contexts. A study conducted in Ukraine and Georgia among undergraduates and graduates of the Faculty of Business management reported lower level of readiness towards online learning [16]. Similarly low level of readiness was reported by Japanese undergraduates in the field of humanities, science and engineering [27]. Whereas high level of readiness towards online learning was reported by nursing students [23] and undergraduate dental students in Saudi Arabia [25]. Similarly, university students in Turkey reported high level of overall online readiness [19]. One possible explanation for these divergent findings may be due to the use of different instruments for measuring online readiness amongst this population. As there are no universally standardised instruments, a plethora of instruments were reported in the literature. This makes it challenging to compare findings between studies and highlight the need for universally standardised instruments to measure online readiness amongst student populations.

The highest score in this study was for equipment readiness. Equipment readiness refers to having equipment or devices needed for online learning such as a computer or smart phone [13, 18]. This finding is comparable to a study among undergraduates in Thailand where 82% and 74% of undergraduates owned smart phones and computers, respectively [28]. Although undergraduates owned their devices and had free internet connection within their educational institutions, this study did not explore the internet connectivity at their residence. In a recent study among university students in Malaysia, it was reported that the students’ readiness on computer and internet self-efficacy was high, however the main challenge was poor internet connectivity [45]. This suggests that poor internet connectivity, outside of their educational institutions, could affect the readiness of the undergraduates for online learning. This is particularly important as previous research indicated that majority of the students (71.4%) accessed their online courses from their hostels, followed by campuses and homes [43]. Due to restrictions imposed in response to the COVID-19 pandemic, most students likely engaged with online learning from their homes and residence, where poor internet connectivity can be a barrier.

The moderate readiness for online learning in our sample is different to students at a public university in Malaysia, who were spending an average of 6 h/day on their smartphones [46]. This may be because smartphones are not exclusively used for educational purposes [27] and higher technical skills (navigation, ICT skills and handling minor technical issues) are required when using online systems [47]. In our study, despite having information technology experience, being tech savvy and possessing higher level of equipment readiness, the undergraduates’ technical competencies to online readiness was still moderate. Rahim et al. [48] did report high equipment readiness in terms of possession of computers and high technological readiness for majority of the internet skills amongst other Malaysian students. However, these findings were limited to internet skills and the sample in [48] study did not include any students from health science background.

The reason for moderate technical readiness amongst physiotherapy undergraduates in our study is unclear but could be due to lack of familiarity, lack of motivation or skills to utilize online tools for learning purposes [26]. Although the current generation is known to be techno-dextrous [24], lack of technical skills specifically about online learning might be one possible explanation. This could have negative implications [22, 26] and limited engagement [20]. Enabling measures such as providing a preparatory course for online learning [20] and customised courses to suit undergraduates could be possible solutions.

Higher scores in social competencies with instructor and classmates are associated with higher scores in motivation [17]. Hence, enhancing social competencies may enhance psychological readiness towards online learning. Moderate level of social competencies among undergraduates in our study is consistent with a similar study in the Malaysian context [12]. Social competencies is a vital skill [14], especially for communication and coordination in the online learning environment [49]. It measures self-efficacy for social interaction with educators and with peers [14], and social presence can enhance the effectiveness of instruction in online learning environment [50]. Online learning based on constructivist pedagogy encourages learners to be engaged with others online to enhance collaboration [51]. Sharing knowledge through participation and social interaction is beneficial for knowledge procurement in online learning settings [52]. Therefore, as many online courses are underpinned by constructivism pedagogy [53], which focus on student’s communicative competency with both instructor and course mates [54], social competency for online learning is critical.

Other factors such as gender and institution had no impact on the level of readiness among undergraduates in our study. Similarly, in previous Malaysian studies, and studies across the globe, gender [1, 15, 48, 55, 56], institution [15], ethnicity and level of education [57] had no influence for online readiness, although these researches did not exclusively focus on undergraduates.

There are some limitations to our study that should be taken into consideration for application and generalizability of the findings. Only undergraduates from physiotherapy courses were involved in this study and the online readiness domains evaluated were limited to equipment readiness, technical competencies, social competencies with instructor and classmates and communication competencies. Other factors such as socio-economic, psychological readiness, self-efficacy [12] and budget readiness [44] should be explored in future studies about online learning readiness. This study was conducted prior to the COVID-19 pandemic and during this time, the sample in this study may have been opportunistically exposed to a range of online teaching and learning initiatives (lecture or practical-based or flipped classroom teaching and learning using technology, digital platforms and online resources). While this may be confounded how the students responded in this study, it is also likely that post COVID-19 pandemic, the undergraduates’ readiness may have altered given the increased focus on, and greater reliance on, online teaching and learning initiatives. Using a mixed method study design, qualitative research could shed important insights on the “why” and help to explore and unpack the nuances related to how well students are ready, or not, to engage with online learning. Finally, future research could explore educator’s readiness for online teaching and how it influences student’s readiness towards online learning.

## Conclusion

Physiotherapy undergraduates in the present study reported moderate to high levels of readiness towards online learning. While there were no differences in readiness level based on gender and institution, in comparison to senior physiotherapy undergraduates, junior students had higher level of social competencies with instructor. However, this could be further strengthened through preparatory courses or targeted training to cater for different levels of readiness amongst student cohorts. Tailored online learning strategies, which cater for students’ needs, can be implemented to improve the experience of, and impacts from, online learning.

## Data Availability

The data and materials are not available for open access, since their access is bound by the ethical agreement approved by the institutions and made with the physiotherapy undergraduates in this study.

## Abbreviations

SOLR: Student Online Learning Readiness Questionnaire
COVID 19: Coronavirus disease 2019

## References

[1] Agius B (2004). Studetns readiness for online learning: a case study from the Faculty of Education, University of Malta. J Malt Educ Res.

[2] Higgs JOY, Hunt A, Higgs C, Neubauer D (1991). Physiotherapy education in the changing international healthcare and educational contexts. Adv Physiother.

[3] Bennett S, Maton K, Kervin L (2008). The ‘digital natives’ debate: a critical review of the evidence. Br J Educ Technol.

[4] Siemens G (2004). Connectivism: a learning theory for the digital age.

[5] AlHaqwi AI, Taha WS (2015). Promoting excellence in teaching and learning in clinical education. J Taibah Univ Med Sci.

[6] Dhawan S (2020). Online learning: a panacea in the time of COVID-19 crisis. J Educ Technol Syst.

[7] Ellman MS, Schwartz ML. Online learning tools as supplements for basic and clinical science education. Journal of medical education and curricular development, *3*, JMECD.S18933. 2016;3:JMECD.S18933. 10.4137/JMECD.S18933. 10.4137/JMECD.S18933 PMC5736292 29349323

[8] Hammarlund CS, Nilsson MH, Gummesson C (2015). External and internal factors influencing self-directed online learning of physiotherapy undergraduate students in Sweden: a qualitative study. J Educ Eval Health Prof.

[9] Adebisi TA, Oyeleke O (2018). Promoting effective teaching and learning in online environment: a blend of pedagogical and Andragogical models. Bulgarian J Sci Educ Policy (BJSEP).

[10] Hossain MS, Shofiqul Islam M, Glinsky JV, Lowe R, Lowe T, Harvey LA (2015). A massive open online course (MOOC) can be used to teach physiotherapy students about spinal cord injuries: a randomised trial. J Phys.

[11] Thang SM, Bidmeshki L (2010). Investigating the perceptions of UKM undergraduates towards an English for science and technology online course. Comput Assist Lang Learn.

[12] Subramaniam TT, Suhaimi NAD, Latif LA, Abu Kassim Z, Fadzil M (2019). MOOCs readiness: the scenario in Malaysia. Int Rev Res Open Distrib Learn.

[13] Coopasami M, Knight S, Pete M (2017). e-learning readiness amongst nursing students at the Durban University of Technology. Health sa gesondheid.

[14] Yu T, Richardson JC (2015). An exploratory factor analysis and reliability analysis of the student online learning readiness (SOLR) instrument. Online Learning.

[15] Atousa R, Rahbania Z, Attaran M (2016). Students’ readiness for E-learning application in higher education. Malaysian Online J Educ Technol.

[16] Blayone TM, Olena, Kokhan, Marianna, Kavtaradze, Medea, et al. Profiling the digital readiness of higher education students for transformative online learning in the post-soviet nations of Georgia and Ukraine. Int J Educ Technol High Educ. 2018;15(37). 10.1186/s41239-018-0119-9.

[17] Bovermann K, Weidlich J, Bastiaens T. Online learning readiness and attitudes towards gaming in gamified online learning – a mixed methods case study. Int J Educ Technol High Educ. 2018;15(1). 10.1186/s41239-018-0107-0.

[18] Bsaol G, Cigdem H, Unver TK (2018). Variables Explaining the Online Learning Readiness Level of Students: Turkish Vocational College Example. Eur J Educ Stud.

[19] Caliskan S, Tugun V, Uzunboylu H (2017). University Students’ readiness for E-learning. ENSAYOS, Revista de la Facultad de Educación de Albacete.

[20] Kołodziejczak B, Roszak M (2017). ICT competencies for academic E-learning. Preparing students for distance education - authors’ proposal. Int J Inform Comm Technol Educ.

[21] Kpolovie PJ, Iderima EC (2016). Readiness for MOOCS: learners’ inequity in Nigeria. EPRA Int J Econ Bus Rev.

[22] Panuwatwanich K, Stewart R (2012). Linking online learning readiness to the use of online learning tools: the case of postgraduate engineering students. Paper presented at the AAEE2012 conference, Melbourne, Australia.

[23] Wafaa G, Ali M. Nursing students’ readiness for e-learning experience. Gynecol Obstetr. 2016;6(6). 10.4172/2161-0932.1000388.

[24] Adams DS, Bambang, Mohamed A, Noor NSM (2018). E-learning readiness among students of diverse backgrounds in a leading Malaysian higher education institution. Malays J Learn Instr.

[25] Asiry MA (2017). Dental students’ perceptions of an online learning. Saudi Dental J.

[26] Linjawi AI, Alfadda LS (2018). Students’ perception, attitudes, and readiness toward online learning in dental education in Saudi Arabia: a cohort study. Adv Med Educ Pract.

[27] Mehran P, Alizadeh M, Koguchi TH (2017). Are Japanese digital natives ready for learning english online? A preliminary case study at Osaka University. Int J Educ Technol High Educ.

[28] Ngampornchai A, Adams J. Students’ acceptance and readiness for E-learning in northeastern Thailand. Int J Educ Technol High Educ. 2016;13(1). 10.1186/s41239-016-0034-x.

[29] Chow SH, Ng F, Mat SC (2007). An investigation on e-learning readiness of engineering students. Instit Eng Malaysia.

[30] Kaur K, Abas ZW (2004). An assessment of e-learning readiness at Open University Malaysia. Paper presented at the international conference on computers in education 2004.

[31] Goode P (2018). Procedure for cannulating a Dialysis access: using the ASSURE model and Gagne’s events of instructions.

[32] Brook, R. L. (2014). Using the ADDIE model to create an online strength training program: an exploration. (doctor of philosophy in curriculum and instruction), Virginia Polytechnic Institute and State University https://vtechworks.lib.vt.edu/handle/10919/47431?show=full

[33] Hsu TC, Lee-Hsieh J, Turton MA, Cheng SF (2014). Using the ADDIE model to develop online continuing education courses on caring for nurses in Taiwan. J Contin Educ Nurs.

[34] Tobase L, Peres HHC, Almeida DM, Tomazini EAS, Ramos MB, Polastri TF (2017). Instructional design in the development of an online course on basic life support. J Sch Nurs Univ Sao Paulo.

[35] Akbulut Y (2007). Implications of two well-known models for instructional designers in distance education:dick-Carey versus Morrison-Ross-Kemp. The Turkish Online J Distance Educ.

[36] Obizoba C (2015). Instructional design model framework for innovative teaching and learning methodologies.

[37] Lozano LM, García-Cueto E, Muñiz J (2008). Effect of the number of response categories on the reliability and validity of rating scales. Methodol Eur J Res Methods Behav Soc Sci.

[38] Yu T. Examining construct validity of the student online learning readiness (SOLR) instrument using confirmatory factor analysis. Online learning. 2018;22(4). 10.24059/olj.v22i4.1297.

[39] Evans SJW (1991). Go for small random samples with high response rates. BMJ.

[40] Prince M, Wright P, Stern J, Phelan M (2012). 9 - Epidemiology. Core psychiatry.

[41] Barde MP, Barde PJ (2012). What to use to express the variability of data: standard deviation or standard error of mean?. Perspect Clin Res.

[42] Khalid N, Zainuddin N (2020). A mixed method study on online learning readiness and situational motivation among mathematics students using gamified learning objects. ISLĀMIYYĀT.

[43] Embi MA (2011). e-Learning in Malaysian Higher Education Institutions: Status, Trends, & Challenges: Department of Higher Education Ministry of Higher Education.

[44] Hussin S, Manap MR, Amir Z, Krish P (2012). Mobile learning readiness among Malaysian students at higher learning institutes. Can Center Sci Educ.

[45] Chung E, Noor NM, Mathew VN (2020). Are you ready? An assessment of online learning readiness among university students. Int J Acad Res Progress Educ Dev.

[46] Zulkefly SN, Baharudin R (2009). Mobile phone use amongst students in a University in Malaysia: its correlates and relationship to psychological health. Eur J Sci Res.

[47] Oketch HA, Njihia JM, Wausi AN (2014). E-learning readiness assessment model in Kenyas’ higher education institutions: a case study of University of Nairobi. Int J Sci Knowledge.

[48] Rahim, N. M., Yusoff, S. H. M., & Latif, S. A. (2014). Assessing students’ readiness towards e-learning. 750-755. AIP conference proceedings 1605, 750 (2014); published online: 17 February 2015. doi: 10.1063/1.4887684.

[49] Olaf Z-R, Brindley CWJE, Zawacki-Richter O (2004). The growing importance of support for learners and Faculty in Online Distance Education. Learner support in open, online and distance learning environments: Bibliotheks- und Informationssystem der Universität Oldenburg.

[50] Krish P, Maros M, Stapa SH (2012). Sociocultural factors and social presence in an online learning environment. GEMA Online™ J Lang Stud.

[51] Ruey S (2010). A case study of constructivist instructional strategies for adult online learning. Br J Educ Technol.

[52] Ma W, Yuen A, Tsang P, Cheung SKS, Lee VSK, Huang R (2010). Understanding Online Knowledge Sharing: An Exploratory Theoretical Framework. Hybrid Learning. ICHL 2010. Lecture notes in computer science.

[53] Hamat A, Embi MA (2010). Constructivism In The Design Of Online Learning Tools.

[54] Syed-Mohamad SM, Pardi KW, Zainal NA, Ismail Z (2006). Expanding nursing education through e-learning: a case study in Malaysia. Stud Health Technol Inform.

[55] Suttiwan T (2013). Students’ readiness for E-learning: a case study of Sukhothai Thammathirat Open University, Thailand. J Learning Higher Educ.

[56] Gay GHE (2018). Fixing the ‘Ready’ in E-Learning Readiness.

[57] Aminul I, Rahim NAA, Liang TC, Momtaz H (2011). Effect of demographic factors on E-learning effectiveness in a higher learning institution in Malaysia. Int Educ Stud.

